# Molecular Dynamics Simulations of NXT-Modified Silica Dispersion Mechanism in Natural Rubber

**DOI:** 10.3390/polym17243237

**Published:** 2025-12-05

**Authors:** Chunmei Lv, Fei Niu, Rongfeng Jiang, Yuan Hu, Lu Liu, Xiaolai Zhang

**Affiliations:** 1 School of Chemistry and Chemical Engineering, Shandong University, Jinan 250100, China; 202312237@mail.sdu.edu.cn; 2 Tongli TyRE Co., Ltd., Huaqin Industrial Park, Yanzhou District, Jining 272100, China; fei.niu@hixih.com.cn (F.N.); rongfeng.jiang@hixih.com.cn (R.J.); yuan.hu@hixih.com.cn (Y.H.); lu.liu@hixih.com.cn (L.L.)

**Keywords:** molecular dynamics simulations, natural rubber, NXT, silica, surface modification, dispersibility, tensile deformation

## Abstract

To tackle the critical challenges of silica dispersion and interfacial compatibility in natural rubber composites, this study investigated the dispersion behavior of 3-Octanoylthio-1-propyltriethoxysilane (NXT)-modified silica in natural rubber (NR) and the mechanism by which it affects mechanical properties. Three distinct models were constructed: an NR model, an NR composite model containing unmodified silica (SiO_2_), and an NR composite model containing NXT-modified silica (NXT-SiO_2_). The radial distribution function (RDF) was used to characterize the dispersion of fillers. The results of filler–filler interactions revealed a reduction in the number of hydrogen bonds between NXT-SiO_2_ fillers, weakening the filler network strength and enabling NXT-SiO_2_ to exhibit excellent dispersion. The results of filler–rubber interactions indicated that NXT-SiO_2_ exhibited stronger interaction forces and compatibility with natural rubber compared to SiO_2_. To verify the effect of NXT-SiO_2_ on the mechanical properties of natural rubber composites, uniaxial tensile deformation via molecular dynamics simulation was performed on the three models. The simulation results show that the addition of NXT-SiO_2_ significantly increases the tensile strength and fracture strain of the composite material, markedly enhancing its mechanical properties. Further studies indicate that NXT-SiO_2_ improves the overall mechanical properties of the material by altering the distribution of local natural rubber chains. This work elucidated the intrinsic mechanisms—on a molecular level—by which NXT silane coupling agent modifications enhance the dispersion of fillers and improve the mechanical properties of rubber, thereby providing a theoretical basis for the design of high-performance rubber composites.

## 1. Introduction

Natural rubber is one of the most commonly used elastomers in tire production [1,2]. To enhance the performance of composite materials, fillers are typically added for reinforcement [3,4,5], with carbon black and silica being the most commonly used reinforcing fillers [6,7,8,9,10,11]. With the development of “green tires”, silica has proven to be the preferred filler for manufacturing high-performance tires [12,13]. However, the inherent chemical dissimilarity between silica and natural rubber, characterized by the former’s polar surface and the latter’s non-polar backbone, typically results in poor compatibility in unmodified systems. Additionally, while external factors such as particle size and processing conditions can influence agglomeration, the primary mechanism driving silica clustering remains the hydrogen bonding between its surface polar hydroxyl groups. The abundance of these groups consequently results in poor dispersion within the non-polar rubber matrix. This undermines filler–rubber interfacial interactions and adversely affects the rubber compound’s processing characteristics and mechanical properties [14,15]. Therefore, it is necessary to enhance the dispersion and compatibility of silica fillers in natural rubber and optimize the interfacial interaction between them.

To improve the performance of silica fillers, a common method is to modify the silica surface by adding silane coupling agents, which can significantly enhance the interfacial properties between silica and polymers [16,17,18,19]. Surya et al. [20] reported that TESPT increased the minimum torque of silica-filled ENR compounds and affected the degree of silica dispersion. You et al. [21] reported that Si69 was grafted onto the surface of silica, thereby significantly improving the water contact angle and enhancing the mechanical properties of modified silica/natural rubber composites. The silane coupling agent modification method not only effectively reduces the abundance of hydroxyl groups on the silica surface, enhancing its dispersion in natural rubber, but also significantly improves the interface compatibility between silica and the natural rubber matrix [22,23]. 3-Octanoylthio-1-propyltriethoxysilane (NXT) is a commonly used bifunctional organosilane coupling agent in the rubber industry. Compared to other coupling agents, the octanoyl group as a blocking group of NXT silane results in the lower reactivity of silane during the mixing process, and NXT exhibits higher scorch safety [24,25]. However, the mechanisms by which NXT silane coupling agents enhance the dispersion of silica within rubber matrices and consequently improve mechanical properties remain poorly understood at the molecular level.

With the advancement of computational chemistry, molecular dynamics (MD) simulations have become a powerful tool for investigating the microscopic structure and properties of materials. At the molecular level, molecular dynamics simulations have been used to explain interactions between interfaces, reveal the interface mechanism between fillers and rubber molecular chains, and have also been proven to be an effective means of evaluating the mechanical properties of polymer materials [26,27]. Wang et al. [28] employed molecular dynamics (MD) simulations to investigate the thermomechanical and tribological properties of graphene–nano-silica (GNS)-reinforced natural rubber (NR) and discussed the intrinsic interactions and wear mechanisms within polymer nanocomposites at the atomic scale. Liang et al. [29] employed molecular dynamics simulations to investigate the thermal conductivity of natural rubber (NR) and polybutadiene (PB) under varying temperature and pressure conditions, in addition to their interfacial heat transfer characteristics. Long et al. [30] comprehensively investigated the interaction behavior between natural rubber and lignin (NR-L) through molecular dynamics simulations. Liu et al. [31] employed molecular dynamics simulations to study the mechanism of enhancing the mechanical and tribological properties of nitrile rubber by adding nano-SiO_2_ at the molecular level. Jiang et al. [32] investigated the interfacial interaction mechanisms between SBR and GTR through tensile and shear behavior studies. Wang et al. [33] used molecular dynamics methods to establish a SiO_2_/cellulose model, grafting silane coupling agents onto the SiO_2_ surface to enhance the interfacial bonding strength between the filler particles and the matrix and thereby promoting close molecular bonding. The enhanced interfacial bonding reduces the thermal motion of the molecular chains, thereby improving the thermal stability and mechanical properties of the composite material. Therefore, using molecular dynamics simulation methods to study the interaction between rubber and fillers has become a feasible approach.

In this study, NR, NR/SiO_2_, and NR/NXT-SiO_2_ composite material models were constructed via MD simulations. This simulation focuses on equilibrium conditions, emphasizing the physical dispersion of fillers during the initial stage of compounding. The objective is to elucidate the mechanism governing the interactions of modified fillers within the natural rubber matrix. Mixing conditions, such as shear and complex temperature profiles, were not considered. In the investigated system, covalent bonds formed through silane coupling reactions between rubber and fillers have not yet been established at this stage [34,35,36]. Through molecular dynamics simulations, the mechanism by which NXT-modified silica enhances its dispersion and improves the mechanical properties of natural rubber at the molecular scale is evaluated. Radial distribution function (RDF) analyses substantiated the enhanced dispersion uniformity of NXT-SiO_2_ fillers within the rubber matrix. The interactions between filler–filler and filler–rubber interfaces were analyzed using hydrogen bond analysis, mean square displacement (MSD), free volume fraction (FFV), independent gradient model (IGM), and interaction energy calculations. Uniaxial tensile deformation was used to simulate the tensile process of the composite material, elucidating the mechanism by which modified fillers enhance mechanical properties and providing a theoretical basis for the design of high-performance rubber composites.

## 2. Simulation Methods and Details

### 2.1. Construction of the Simulation System

**Figure 1:**
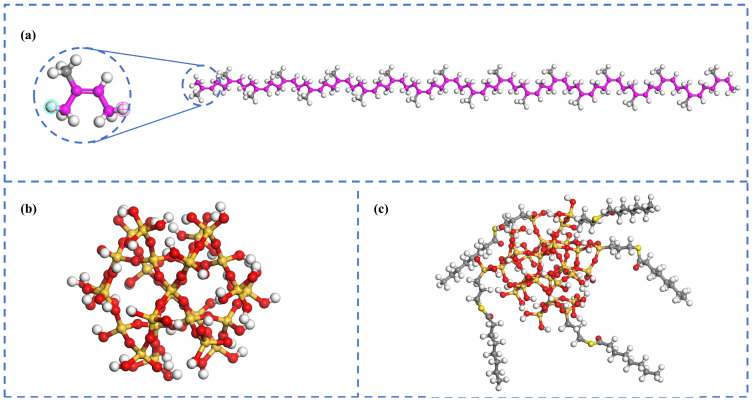
Molecular model diagram: (**a**) NR; (**b**) SiO_2_; (**c**) NXT-SiO_2_. Atomic color scheme is assigned as follows: H (white), C (gray), O (red), Si (orange), and S (yellow). Carbon atoms in the natural rubber backbone are colored purple herein for clarity; all subsequent figures depict carbon in gray for consistency, while other atomic colors remain unchanged.

In molecular dynamics simulations, the construction of the model is critical to the simulation results. The monomer of natural rubber is 1,4-isoprene. Existing research indicates that the molecular chain of natural rubber can already exhibit the characteristics of a polymer chain at a polymerization degree of 20 [37,38]. Therefore, we used Materials Studio 2020 software to construct a non-crosslinked natural rubber molecular chain with a polymerization degree of 20. We represented silica fillers (SiO_2_) using spherical particles with a radius of 0.6 nm, and hydroxyl groups were introduced to eliminate the unsaturated boundary effect and impart genuine surface polarity. This treatment is crucial for accurately simulating the interfacial interactions between natural rubber and silica, as well as the agglomeration behavior of the filler. The silica filler’s surface underwent a typical silanization reaction with the NXT silane coupling agents. Accordingly, we developed a model of silica fillers modified with the NXT molecules (NXT-SiO_2_) [39]. The molecular models are shown in Figure 1.

**Figure 2:**
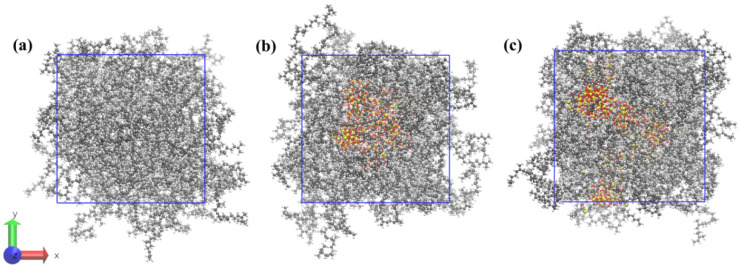
Composite models of each system after balancing: (**a**) NR; (**b**) NR/SiO_2_; (**c**) NR/NXT-SiO_2_.

Three simulation systems were constructed, each containing 50 NR molecular chains. Two of these systems were supplemented with SiO_2_ and NXT-SiO_2_, respectively. First, the steepest descent method was used to minimize the energy of all composite models to eliminate unreasonable configurations. The systems were first equilibrated for 10 ns in the NVT (constant number of particles, volume, and temperature) ensemble. Subsequently, a 12 ns annealing simulation was conducted in the NPT (constant number of particles, pressure, and temperature) ensemble. Finally, the temperature was reduced to 298 K over 1 ns under the NPT ensemble, followed by a 200 ns run in the same ensemble. The total energy curves of the system converge and gradually flatten with increasing simulation time, indicating that the system of the model has reached equilibrium (Figure S1) [37,40]. The resulting composite models are shown in Figure 2. The parameters of each system are listed in Table 1. Molecular dynamics simulations reveal that the radii of gyration (R_g_) of the polymer chains in the three models are 26.93, 28.97, and 30.37 Å, respectively, all exceeding the radius of silica nanoparticles (6 Å) [41,42,43]. Consequently, when silica content is constant and the rubber chain length exceeds the length required to achieve fundamental polymer dynamics, the interaction between silica filler and natural rubber is primarily determined by the chemical structures of the filler and natural rubber. Therefore, the construction of the relevant molecular model is reasonable. In addition, the final density of the pure NR model was 0.91 g/cm^3^, which is consistent with the true density of the natural rubber polyisoprene of 0.92 g/cm^3^ [28].

### 2.2. Molecular Dynamics Simulation Details

The simulation carried out in this study was completed using the GROMACS 2022 software package [44], with the GROMOS54a7 force field [45]. The force field files for the simulated molecules were generated online using the ATB (Automatic Topology Builder) tool [46]. The potential energy functions included bonding interactions such as bond length, bond angle, and dihedral angle, as well as Coulombic interactions and Lennard–Jones potentials:

(1) $$U_{ij}(r)=4\varepsilon_{ij}\left[{\left(\frac{\sigma_{ij}}{r_{ij}}\right)}^{12}-{\left(\frac{\sigma_{ij}}{r_{ij}}\right)}^{6}\right]+\frac{q_{i}q_{j}}{r_{ij}}$$

where r_ij_ is the distance between the i-th and j-th atoms, q_i_ is the charge of the i-th atom, σ_ij_ is related to the equilibrium distance between the i-th and j-th atoms, and ε_ij_ is the interaction strength.

Periodic boundary conditions were applied in all three dimensions of the simulation box. The simulation temperature under the NVT ensemble is controlled at 500 K. During the NPT annealing process, the composite model is subjected to five cycles of annealing at 500 K to 200 K to systematically eliminate unreasonable configurations that may arise from initial structural modeling, thereby rendering the material configuration more rational, which does not represent actual experimental conditions [41]. After annealing, the temperature of the simulated system was lowered to 298 K using the same temperature interval of 25 K and a cooling rate of 25 K/100 ps [47]. A simulation of 200 ns was then performed under NPT conditions at 298 K. The simulation process used the V-rescale thermostat method [48] to control the temperature, and the Berendsen method [49] was used for pressure control with a size of 1 atm. The particle mesh Ewald (PME) method [50] was used to calculate long-range electrostatic interactions, and the truncation method was used to calculate the van der Waals interactions and to take into account the long-range corrections to the van der Waals interactions with a truncation radius of 1 nm. During the simulation, the LINCS algorithm was used to constrain all bonds connected to hydrogen atoms [51]. The simulation employed a simulation step size of 1 fs, with trajectories saved at a frequency of one frame every 5 ps. After the simulation calculation was completed, the visualization of the trajectory was generated using the VMD software 1.9.4 [52].

## 3. Results and Discussion

### 3.1. Characterization of Filler Dispersibility

#### 3.1.1. Radial Distribution Function Analysis

The radial distribution function (RDF) of molecules is a physical quantity that characterizes the microstructural properties of materials. It is the statistical average of the number of other particles at a distance r around a particle and is mainly used to analyze the structural state of a simulated system. In molecular simulations, g_AB_(r) can be used to characterize the dispersion of fillers [42,53]. The calculation of g_AB_(r) is shown in the following formula (in this study, particle A and particle B both represent the center of mass of the fillers within the same system. Here, A represents the center of mass of the reference group):

(2) $$g_{AB}\left(r\right)=\frac{\left\langle n_{AB}\left(r\right)\right\rangle }{4\pi r^{2}\Delta \rho_{AB}}$$

where n_AB_(r) is the average number of atom pairs between r and r + Δr, and ρ_AB_ is the average density of particle type B relative to particle type A.

**Figure 3:**
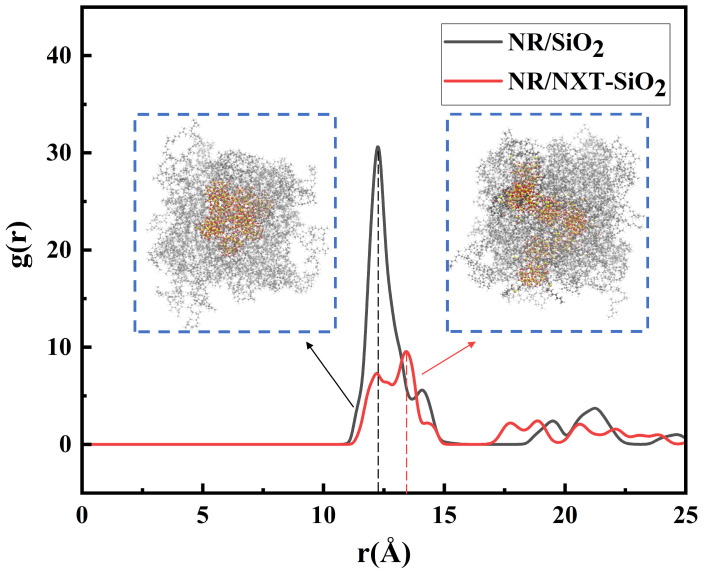
Radial distribution function between the molecular centers of mass of the fillers.

The dispersion of the fillers is determined by calculating the radial distribution function between the molecular centers of the fillers. In this study, the RDF between the centers of mass of the filler molecules in the NR/SiO_2_ and NR/NXT-SiO_2_ composite systems was calculated, with the results shown in Figure 3. The peak of g(r) indicates a high probability that the distance between two fillers falls within this range. A high and sharp first peak indicates a tendency for filler agglomeration. In contrast, the composite with NXT-SiO_2_ exhibits a significantly reduced peak intensity and a flatter g(r) profile, which signifies a more homogeneous, random dispersion state of the NXT-SiO_2_ fillers within the natural rubber matrix. At the same time, the authors of [42] also report that the larger the r value at the maximum peak, the greater the distance between the silica fillers, and the better the dispersion. It can be observed that the distance corresponding to the maximum peak in the NR/SiO_2_ system is smaller than that in the NR/NXT-SiO_2_ system. This indicates that the dispersion of NXT-SiO_2_ fillers in natural rubber is better than that of SiO_2_ fillers. This result is consistent with the observations made by Yang et al. using scanning/transmission electron microscopy (SEM/TEM): no significant agglomeration of fillers was observed in NXT-modified silica, indicating its excellent dispersion properties [54].

#### 3.1.2. Atomic Density Distribution

**Figure 4:**
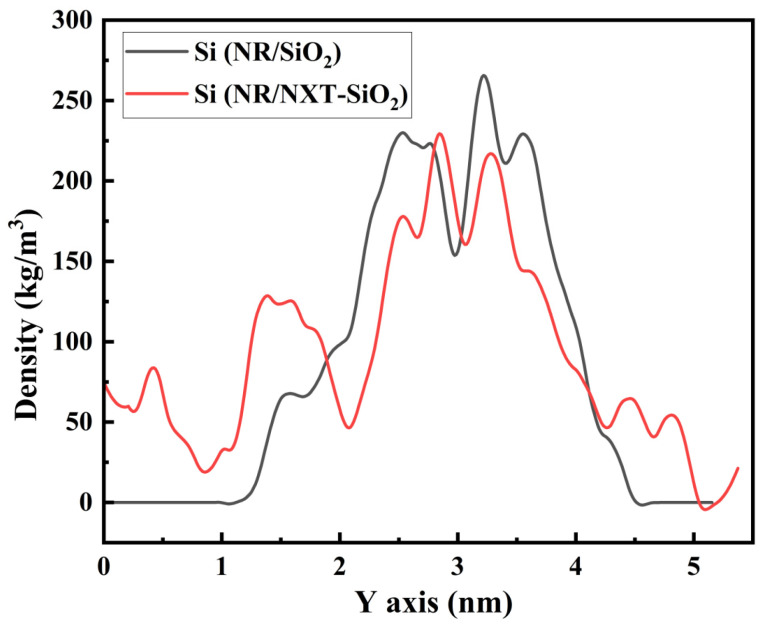
Density distribution of Si atoms along the *y*-axis in the filler molecules.

Atomic density analysis enables the characterization of filler molecules’ dispersion within natural rubber matrices. The density distribution of silicon atoms in filler molecules along the *y*-axis is shown in Figure 4. As observed in the figure, there is a relatively long region with zero density for silicon atoms in the NR/SiO_2_ system, and in another region, their atomic density is relatively high, indicating that the filler may be aggregated in this area. Compared to the NR/SiO_2_ system, the density distribution of silicon atoms in the NR/NXT-SiO_2_ composite system is more uniform, indicating that the dispersion of the modified filler NXT-SiO_2_ is better, with the characteristic peak observed in the RDF in Figure 3 and the visualization in the images confirming this result.

### 3.2. Filler–Filler Interactions

#### Analysis of Hydrogen Bonding Interactions

**Figure 5:**
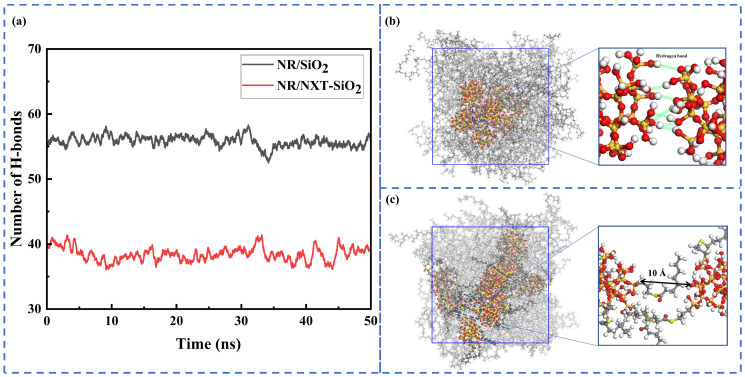
Hydrogen bonding interactions: (**a**) changes in the number of hydrogen bonds between fillers over time; (**b**) localized enlargement of the SiO_2_ fillers; (**c**) localized enlargement of the NXT-SiO_2_ fillers.

The surface of silica contains a large number of polar hydroxyl groups, which tend to aggregate under the influence of hydrogen bonds. This study calculated the number of hydrogen bonds between SiO_2_ and NXT-SiO_2_ fillers over time. Hydrogen bonds were identified using the default geometric criteria of GROMACS: the distance R_DA_ between the donor and acceptor is less than 3.5 Å, and the angle α_HB_ formed between the hydrogen atom donor and acceptor is less than 30°. As shown in Figure 5, it can be observed that the number of hydrogen bonds significantly decreases after modification. This indicates that the improved dispersion of the NXT-SiO_2_ filler in the rubber matrix is due to NXT silane coupling agents undergoing silanization reactions with silica surfaces, fundamentally altering the physicochemical properties of silica surfaces. The reduction in surface hydroxyl groups directly weakens hydrogen bond formation between fillers, effectively inhibiting their aggregation. Upon local magnifications of the filler molecules, it can be observed that SiO_2_ fillers form a larger number of hydrogen bonds, while NXT-SiO_2_ fillers, due to the grafting of NXT silane coupling agents, exhibit reduced surface polarity. Additionally, the presence of the silane coupling agent’s long chain hinders the aggregation of silica molecules, thereby enhancing the dispersion of fillers.

**Figure 6:**
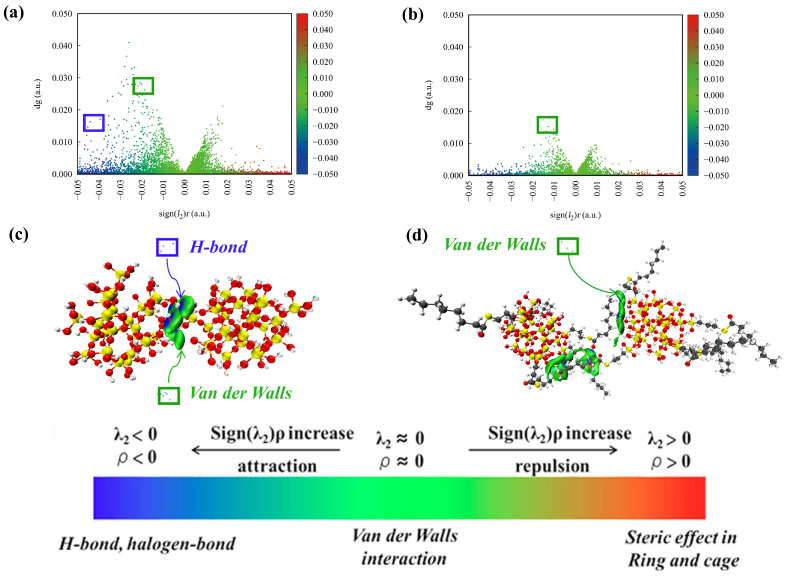
IGM between filler molecules: (**a**,**c**): SiO_2_; (**b**,**d**): NXT-SiO_2_.

The independent gradient model (IGM) enables the qualitative identification and visualization of the types and spatial regions of non-covalent interactions present at the interfaces [55,56]. To better illustrate the interactions between filler molecules, an independent gradient model analysis was performed on the filler molecules using the Multiwfn software 3.8 [57,58]. The isosurface plots and scatter plots are shown in Figure 6. The blue region represents the presence of strong hydrogen-bonding interactions; the green region represents the presence of van der Waals interactions; the red region represents the presence of strong steric hindrance effects. It can be observed in Figure 6 that hydrogen bond interactions and van der Waals interactions exist between SiO_2_ fillers, while NXT-SiO_2_ fillers exhibit van der Waals interactions. Based on IGM visualization analysis, the reason for the improved dispersion of NXT-SiO_2_ is that the modification reduces the hydroxyl groups on the surface of the silica, and the long chains of the grafted silane coupling agents can form van der Waals interactions with the hydroxyl groups on the silica surface, thereby partially shielding them and weakening the ability of the molecules to form hydrogen bonds. Since hydrogen bonds are typically stronger than van der Waals interactions, the attractive forces between SiO_2_ fillers are stronger than those between NXT-SiO_2_ fillers. Therefore, SiO_2_ fillers are more likely to aggregate, while NXT-SiO_2_ fillers have better dispersibility.

### 3.3. Filler–Rubber Interaction

#### 3.3.1. Mean Square Displacement (MSD) Analysis

**Figure 7:**
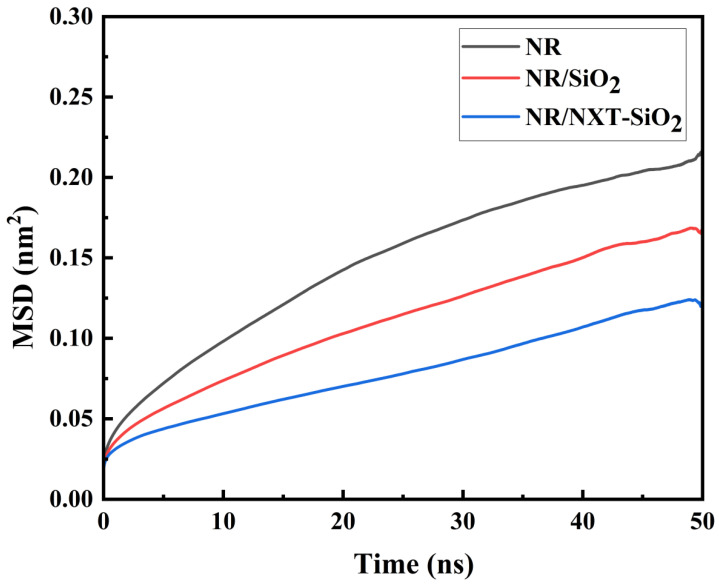
MSD of natural rubber molecular chains.

The mean square displacement (MSD) characterizes the motion trends of molecular chains in polymer composites. It is defined as the distance between a particle’s position at time t and its initial position [59]. As shown in the MSD curves of natural rubber molecular chains at 298 K in different composite models in Figure 7, the MSD of natural rubber molecular chains in the two systems with added filler molecules is smaller than that in the pure natural rubber system, with the lowest MSD observed in the system with added NXT-SiO_2_ fillers. This indicates that filler molecules hinder the movement of natural rubber molecular chains. Compared to SiO_2_, NXT-SiO_2_ exerts a stronger hindering effect on the movement of natural rubber molecular chains, resulting in a lower migration ability. This stronger hindering effect is likely due to a combination of stronger filler–rubber interfacial interactions and a more uniform spatial distribution of the NXT-SiO_2_ filler, both contributing to the more effective restriction of chain mobility [60]. The enhanced hindering effect of filler molecules on natural rubber molecular chains suggests the greater restriction of molecular motion at the atomistic scale, which is often an indicator of potentially improved material stability.

#### 3.3.2. Fractional Free Volume Analysis

Fractional free volume (FFV) characterizes the available movement space and mobility constraints for molecular chains within a material. It is closely correlated with material performance and serves as a critical parameter for predicting trends in the mechanical properties of composites [61]. The formula is as follows:

(3) $$FFV=\frac{V-V_{O}}{V}=\frac{V_{f}}{V}$$

where V_f_ is the volume of the cavity within the system, and V_o_ is the volume occupied by the molecule in the system.

**Figure 8:**
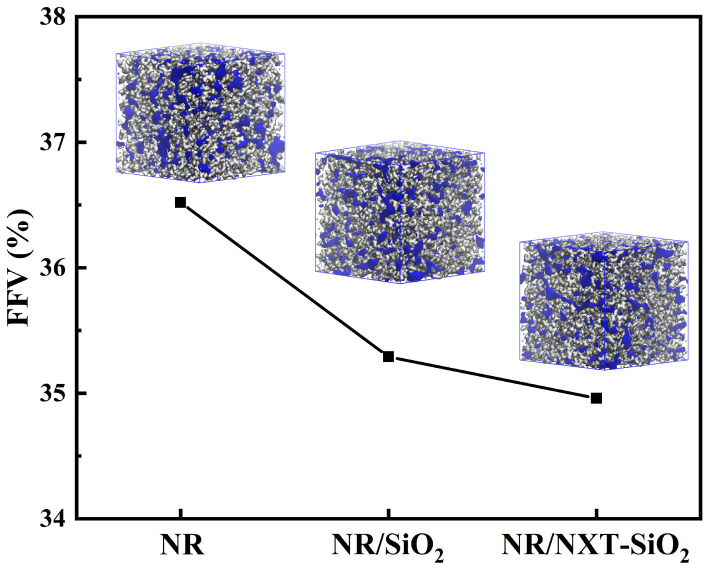
Fractional free volume and Connolly volume morphology.

The FFV of the three models after NPT ensemble equilibration is presented in Figure 8, along with their Connolly volume shapes, where blue and gray denote free volume and occupied volume, respectively. The FFV of the models decreases with the addition of fillers. The FFV of the three composite systems reached 36.52%, 35.29%, and 34.96%. The FFV of the NR/SiO_2_ model is smaller than that of the NR model, and the NR/NXT-SiO_2_ model is the smallest. It can be concluded that in composite systems composed of filler and rubber matrices, the presence of fillers causes molecular chains to pack more tightly, thereby reducing FFV. The NR/NXT-SiO_2_ model shows the most compact structure. The reduced FFV suggests tighter molecular packing, which is expected to enhance stress transfer and potentially improve mechanical properties.

#### 3.3.3. Interaction Energy Analysis

The interaction energy (E_inter_) between natural rubber and fillers in different composite models was calculated. The binding energy (E_binding_), defined as the negative value of the interaction energy between the two components, was also used as a metric to assess compatibility. High E_binding_ values typically indicate strong interfacial adhesion and good compatibility [62]. The calculation formula is as follows:

(4) $$E_{inter}=E_{total}-\left(E_{filler}-E_{NR}\right)=-E_{binding}$$

where E_inter_ represents the interaction energy; E_binding_ represents the binding energy; E_total_ is the total energy of the NR composite system; E_filler_ is the energy of the system containing only the filler; and E_NR_ is the energy of the system containing only NR.

**Figure 9:**
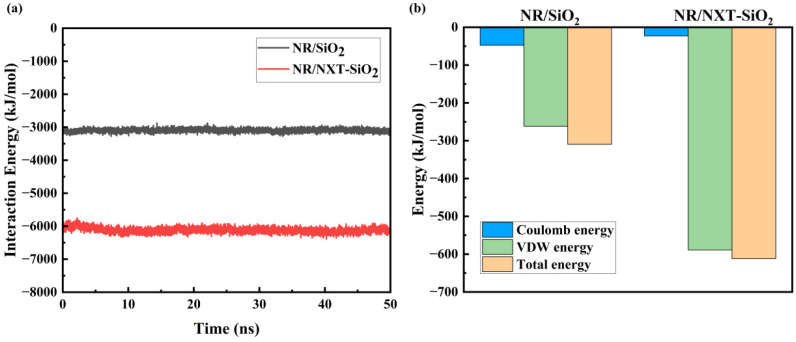
The interaction energy between the filler and natural rubber: (**a**) time-dependent interaction energy; (**b**) interaction energy components between a single filler and natural rubber.

It can be observed in Figure 9a that the E_inter_ between the NXT-SiO_2_ fillers and natural rubber is significantly greater than that between SiO_2_ fillers and natural rubber. The binding energy of the functionalized NXT-SiO_2_ fillers with natural rubber reached 6118.4 kJ mol^−1^, significantly higher than that of the unmodified SiO_2_ fillers (3096.7 kJ mol^−1^). This indicates that after modification, the ability of NXT-SiO_2_ fillers to bind with natural rubber molecules has increased, resulting in more natural rubber molecules aggregating around the NXT-SiO_2_ fillers and the enhanced stability of the composite model. The compatibility between NXT-SiO_2_ fillers and natural rubber is superior to that between SiO_2_ fillers and natural rubber. Additionally, the interaction energy of the final system remains essentially unchanged, indicating that the system has reached equilibrium, which indirectly reflects the feasibility and equilibrium of this computational model. Figure 9b shows the various interaction energies between a filler and natural rubber molecules in different systems. As can be seen, van der Waals interactions are the primary mode of interaction between filler and natural rubber. Additionally, compared to the SiO_2_ filler, the van der Waals interactions between NXT-SiO_2_ fillers and natural rubber are significantly enhanced, while electrostatic interactions are reduced. This typically implies improved interface compatibility between the filler and rubber, as well as enhanced filler dispersion. When van der Waals interactions increase, this indicates that the contact between filler and rubber molecules becomes more intimate, improving interfacial compatibility. The increase in van der Waals interactions also facilitates the dispersion of fillers in rubber, as stronger intermolecular forces promote a more uniform distribution of filler particles within the rubber matrix. Improvements in interface compatibility and filler dispersion generally have a positive impact on the performance of rubber composites, consistent with the results from MSD and FFV, as discussed earlier.

**Figure 10:**
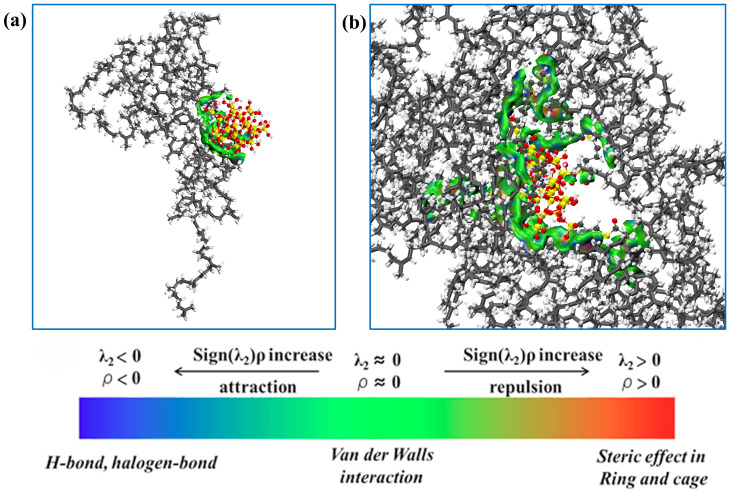
IGM between fillers and NR molecules: (**a**) NR/SiO_2_; (**b**) NR/NXT-SiO_2_.

**Figure 11:**
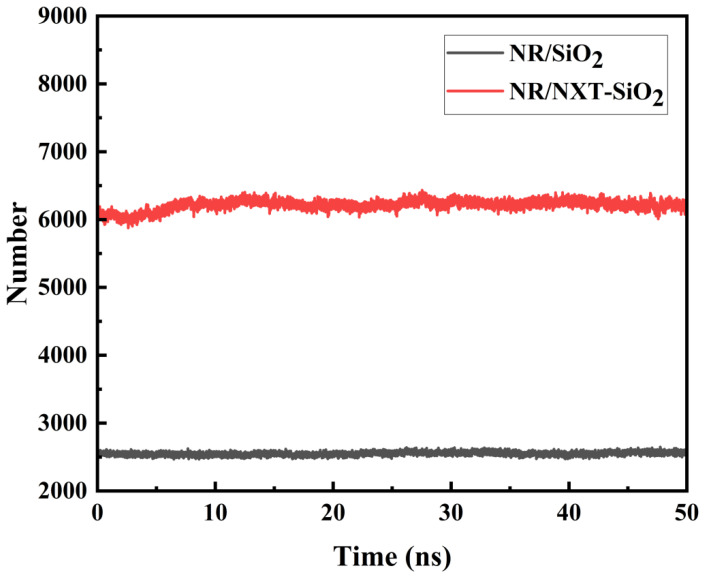
Number of rubber atoms in contact with fillers.

To demonstrate the interactions between filler molecules and rubber molecules, an analysis was conducted using the independent gradient method, with the results shown in Figure 10. The green areas indicate minimal electron density within the weak interaction region, signifying the predominance of dispersion interactions. This distribution demonstrates that van der Waals interactions dominate the binding mechanism at filler–natural rubber interfaces. This conclusion is consistent with the results of the interaction energy calculations. It can be observed that the interaction area between SiO_2_ fillers and natural rubber is smaller than that between NXT-SiO_2_ fillers and natural rubber, indicating that NXT-SiO_2_ fillers can bind with a greater number of natural rubber molecules. This phenomenon is due to SiO_2_ fillers tending to agglomerate, reducing the area available for contact with natural rubber molecules, while NXT-SiO_2_ fillers are more uniformly dispersed in natural rubber. Additionally, the organic long chains grafted onto the silica fillers can interact with natural rubber molecules, enhancing the compatibility between NXT-SiO_2_ fillers and natural rubber. To verify this conclusion, the number of natural rubber atoms in contact with filler molecules in the two composite systems was calculated, as shown in Figure 11. The number of natural rubber atoms in contact with NXT-SiO_2_ is significantly higher than that with SiO_2_, demonstrating that NXT-SiO_2_ can interact more effectively with natural rubber and thereby improve the material’s stability and mechanical properties.

### 3.4. Mechanical Properties During the Stretching Process of Each Model

#### Study of Mechanical Properties During Stretching

**Figure 12:**
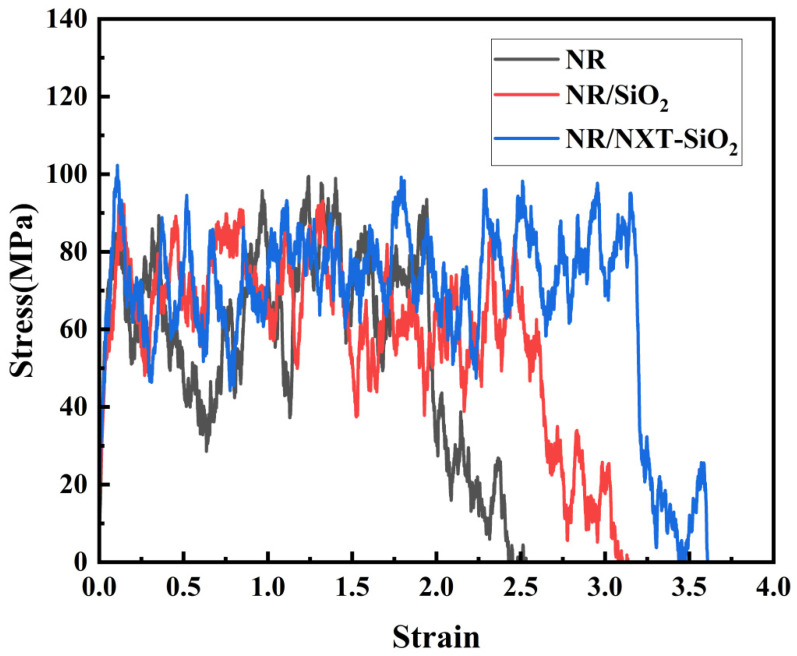
Stress–strain curves of the three models at 298 K.

A uniaxial tensile simulation was performed on the composite model at 298 K with a deformation rate of 0.005 nm·ps^−1^ along the *y*-axis [63]. The stress–strain curves of the three composite models along the *y*-axis are shown in Figure 12. The stress–strain curve of pure natural rubber conforms to the uniaxial tensile characteristic curve of polymer materials, and it is generally consistent with the simulation results [64,65]. Comparing these results with data from the literature reveals that the stress–strain behavior falls within a reasonable range of variation, thereby validating the reliability of using molecular dynamics simulations to calculate tensile behavior [28,47,66]. It can be observed that the addition of SiO_2_ fillers increases the fracture strain ε of the composite material model, indicating that the addition of fillers increases the fracture strain when the composite material is stretched to the point of complete separation at both ends. Additionally, the fracture deformation of the NR/NXT-SiO_2_ system is greater than that of the NR/SiO_2_ system, and the tensile strength is also enhanced. This is because NXT grafting improves the dispersion of fillers in the NR matrix, weakens the filler–filler network, and reduces stress concentration zones. On the other hand, NXT grafting enhances the strength of the filler–rubber interface interaction and suppresses the movement of molecular chains on the filler surface. From the tensile data, it can be seen that improved filler dispersion is beneficial to the mechanical properties of polymer materials, which is consistent with previous results [67].

**Figure 13:**
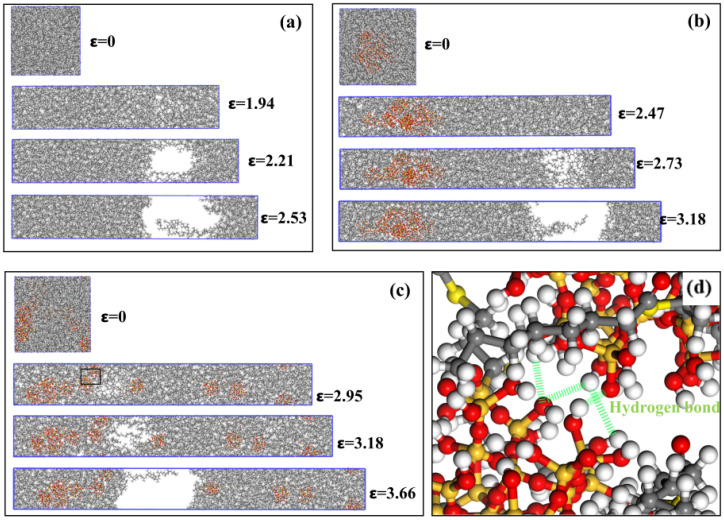
States of various composite models at different stretching stages: (**a**) NR; (**b**) NR/SiO_2_; (**c**) NR/NXT-SiO_2_; (**d**) local enlargement of the NR/NXT-SiO_2_ model, which corresponds to the region designated by the black frame in (**c**).

The states of various composite models at different stages of stretching are shown in Figure 13. Taking the NR model as an example, Figure 13a shows the state diagram of the NR model during the stretching process, where ε = 0 represents the computational model at the initial state of stretching. ε = 1.94 represents the tensile state of the model at the strength limit, after which noticeable fracture voids begin to form within the model. As tensile loading continues, the NR molecular chains begin to loosen at the voids. ε = 2.21 illustrates the gradual increase in fracture voids within the model during the neck-shrinkage fracture stage. During this process, the NR chains at the fracture voids gradually separate, and the tensile stress in the y-direction gradually decreases. ε = 2.53 represents the post-fracture state, where the NR molecular chains spanning the fracture interface are separated beyond the cutoff radius of intermolecular interactions, resulting in zero intermolecular forces between the chains and zero tensile stress. At this point, the NR model is completely ruptured. Figure 13b shows a schematic diagram of the stretching process of the NR/SiO_2_ model. Due to the addition of fillers, the fracture deformation of this model is greater than that of the pure natural rubber model. However, SiO_2_ fillers tend to aggregate, causing stress concentration at the aggregated particles and resulting in uneven stress distributions within the material. The fracture gap appears near the NR molecular chains, distant from the filler distribution. Figure 13c shows a schematic diagram of the stretching process of the NR/NXT-SiO_2_ model. The composite exhibits the highest fracture strain among all tested systems. This superior deformability is attributed to the NXT-SiO_2_ fillers being more uniformly dispersed in the natural rubber matrix. The better the dispersion of the fillers, the greater the stress that the composite material can withstand [68]. Analyses of the fracture interface of the NR/NXT-SiO_2_ model, as shown in Figure 13d, reveal that one side of the fracture interface (left) exhibits stronger interactions due to the formation of numerous hydrogen bonds between fillers, with a tendency to form filler clusters; in contrast, the other side (right) primarily consists of isolated individual fillers. The interface interactions between these isolated fillers and aggregates are relatively weak, making this area a relatively weak connection point in the model. The fracture occurs at this location primarily due to the significant stress concentration points formed at the interface between the clusters and the isolated fillers.

**Figure 14:**
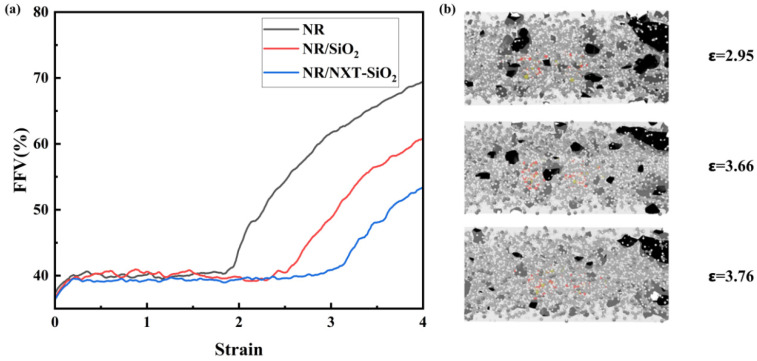
FFV curves and molecular visualization of model surfaces during stretching for each model: (**a**) the variation in the FFV with stretch deformations; (**b**) molecular surface visualization analysis of NXT-SiO_2_ fillers and surrounding natural rubber molecular chains.

To understand the internal conditions of the material during stretching, the variation in the FFV with respect to stretch deformation was plotted [69]. The model’s molecular surface was visualized using the “construct surface mesh” module in the Ovito software 3.10.6 [70]. As shown in Figure 14a, during the initial stage of stretching, the FFV of all three systems increased proportionally. During the yield stage and strengthening stage, the FFV remained in a state of dynamic equilibrium, with little change in internal porosity. As stretching continued, the FFV continued to increase. It can also be observed that the FFV of the system with added NXT-SiO_2_ fillers overall decreases during the stretching process, indicating that the NXT-SiO_2_ fillers render the internal space distribution of the system more uniform during stretching, resulting in more uniform stress distribution in the material. To verify this conclusion, a molecular surface visualization analysis was performed on the NXT-SiO_2_ fillers and the surrounding natural rubber molecular chains. As shown in Figure 14b, during the neck-down fracture stage, while the overall FFV of the model increases, the NR chains around the NXT-SiO_2_ fillers exhibit a decreasing trend, indicating that these fillers locally constrain the polymer matrix and enhance mechanical performance.

**Figure 15:**
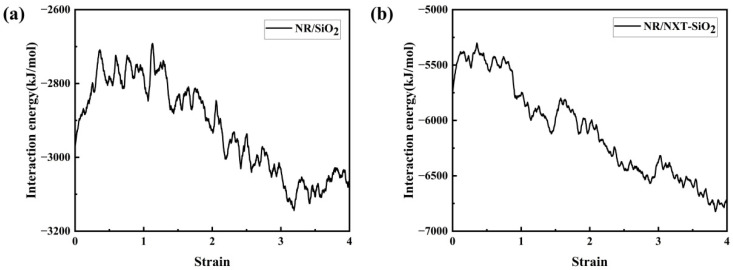
Interaction energy change curve during model stretching: (**a**) NR/SiO_2_; (**b**) NR/NXT-SiO_2_.

The interaction energy between filler molecules and rubber molecules during the stretching process of the two composite models was calculated, with the results shown in Figure 15. It can be observed that since the interaction is negative, a decrease in the absolute value of the interaction energy indicates a reduction in the interaction force. During the elastic stage, the interaction between the two decreases. After entering the yield stage, the interaction between the two increases sharply until the material gradually separates at both ends and breaks, at which point the interaction energy tends to stabilize. It can be observed that in the NR/NXT-SiO_2_ system, the interaction between the fillers and natural rubber molecular chains is significantly stronger than in the NR/SiO_2_ system. Specifically, the NXT-SiO_2_ filler exhibits a stronger ability to hinder the separation of natural rubber molecular chains, thereby enhancing the material’s mechanical properties.

**Figure 16:**
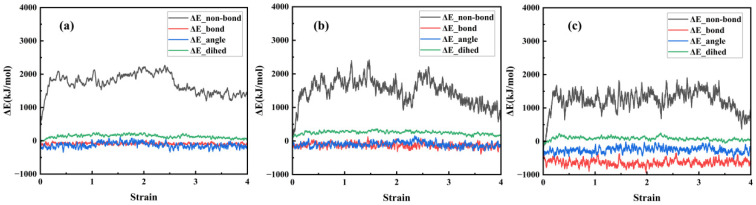
Four energy change curves during the stretching process of three models: (**a**) NR; (**b**) NR/SiO_2_; (**c**) NR/NXT-SiO_2_.

The non-bonded interaction energy, bond energy, angular energy, and dihedral angle energy were calculated for three composite models at 298 K under tensile deformation in the y-direction, with the results shown in Figure 16. The three composite models primarily adjust the system’s energy through non-bonding interaction energy, exhibiting a linear growth trend during the elastic stage and the initial phase of the yield stage. This is primarily due to the sliding mechanism of polymer molecular chains. Non-bonding interaction energy reaches its maximum value during the yield stage and then continuously decreases as the stretching process progresses until the model completely fractures.

## 4. Conclusions

This study comprehensively explores the mechanism by which NXT improves the dispersion of fillers in an NR matrix and enhances the mechanical properties of NR through analyses of filler dispersion, filler–filler interactions, filler–rubber interactions, and tensile simulations. To effectively reveal the core physical mechanisms, our current model employs a fixed set of parameters and component ratios, but we did not systematically investigate the quantitative impact of varying parameters on simulation results. Future research will systematically examine how changes in key parameters affect the macroscopic properties of composite materials. The research findings are as follows:

1. In NXT-SiO_2_ fillers, due to the silanization reaction between NXT silane coupling agents and the silica surface, the physicochemical properties of the silica surface are fundamentally altered. The number of hydroxyl groups on the filler surface is reduced in NXT-SiO_2_ fillers, and the organic long chains of the coupling agent molecules hinder the proximity of hydroxyl groups on the nano-silica surface, reducing the formation of hydrogen bonds between nano-silica particles. This weakens filler–filler interactions, resulting in the improved dispersion of fillers.
2. NXT-SiO_2_ fillers exhibit good dispersion and contain grafted long organic chains. After modification, filler molecules can interact with more rubber molecules; this significantly increases the effective contact area, thereby enhancing the van der Waals interactions that dominate the filler–rubber interface and the interaction between fillers and rubber. Compared to unmodified SiO_2_ fillers, stronger interactions are observed. Additionally, the grafted coupling agent molecules on the surface contain long organic chains, enhancing the compatibility between filler molecules and rubber molecules.
3. Uniaxial tensile deformation simulation of the composite model revealed that the addition of NXT-SiO_2_ fillers enhances the tensile strength limit and increases the fracture limit of the composite material, strengthening its mechanical properties. Analyses showed that, compared to SiO_2_ fillers, the improved dispersibility of NXT-SiO_2_ fillers allows them to distribute uniformly within rubber molecules, facilitating stress transfer. Additionally, it exhibits stronger interaction forces with NR chains, enhancing its ability to prevent NR chain separation. This is the key to improvements in mechanical properties. During the neck-down fracture stage, while the overall FFV of the model increases, the NR chains around the NXT-SiO_2_ fillers exhibit a decreasing trend, indicating that these fillers locally constrain the polymer matrix and enhance mechanical performance.

## Figures and Tables

**Table 1:** Molecular composition and system density in three simulated systems. (“--” indicates that this component is not included in the simulated system).

Simulated System	Number of NR	Number of SiO_2_ Fillers	Number of NXT-SiO_2_ Fillers	Density (g/cm^3^)
NR	50	--	--	0.91
NR/SiO_2_	50	10	--	1.06
NR/NXT-SiO_2_	50	--	10	1.08

## Data Availability

The original contributions presented in this study are included in the article/Supplementary Material. Further inquiries can be directed to the corresponding author.

## Supplementary Materials

The following supporting information can be downloaded at: https://www.mdpi.com/article/10.3390/polym17243237/s1, Figure S1: Total energy of each system as a function of simulation time; Table S1: R_g_ of natural rubber molecular chains in various systems.
