# Supplementary material for: Molecular Dynamics Simulations of NXT-Modified Silica Dispersion Mechanism in Natural Rubber
Source: Polymers (Basel). 2025 Dec 5;17(24):3237. doi: 10.3390/polym17243237 (PMC12736809; doi:10.3390/polym17243237)
Supplement: Supplementary file 1 [file polymers-17-03237-s001.zip › polymers-3952774-supplementary.pdf]

Molecular Dynamics Simulations on NXT-Modified Silica's Dispersion Mechanism in Natural Rubber

Table S1. R_g of natural rubber molecular chains in various systems

Simulated system	NR	NR/SiO_2	NR/NXT-SiO_2
Rg (Å)	26.93	28.97	30.37

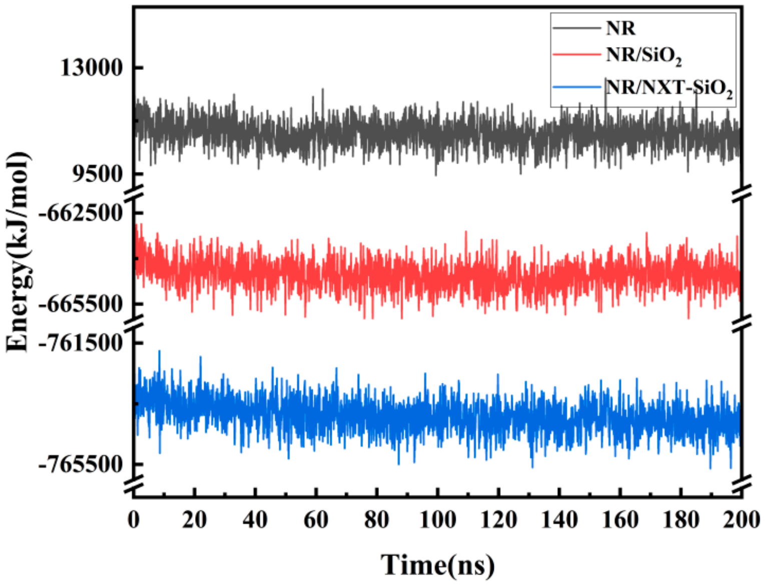

Figure S1. Total energy of each system as a function of simulation time.
